# Cognitive Impairment and Psychological Morbidity Among Stroke Survivors in Rehabilitation: A Cross-Sectional Analysis

**DOI:** 10.3390/jcm14217735

**Published:** 2025-10-31

**Authors:** Ana-Maria Bumbea, Daniela Gabriela Glavan, Ramona-Constantina Vasile, Alexandra Daniela Rotaru-Zavaleanu, Andrei Greșiță, Roxana Surugiu, Sorin Nicolae Dinescu, Irina Burlacu, Madalina Aldea

**Affiliations:** 1 Department of Medical Rehabilitation, University of Medicine and Pharmacy of Craiova, 2 Petru Rares Str., 200349 Craiova, Romania; anamaria.bumbea@umfcv.ro; 2 Department of Psychiatry, University of Medicine and Pharmacy of Craiova, 2 Petru Rares Str., 200349 Craiova, Romania; daniela.glavan@umfcv.ro (D.G.G.); madalina.aldea@umfcv.ro (M.A.); 3 Department of Epidemiology, University of Medicine and Pharmacy of Craiova, 2 Petru Rares Str., 200349 Craiova, Romania; sorin.dinescu@umfcv.ro; 4 Department of Physiology, University of Medicine and Pharmacy of Craiova, 2 Petru Rares Str., 200349 Craiova, Romania; andrei.gresita@umfcv.ro; 5 Department of Biochemistry, University of Medicine and Pharmacy of Craiova, 2 Petru Rares Str., 200349 Craiova, Romania; roxana.surugiu@umfcv.ro; 6 Doctoral School, University of Medicine and Pharmacy of Craiova, 2 Petru Rares Str., 200349 Craiova, Romania; irinamusa23@yahoo.com

**Keywords:** stroke rehabilitation, post-stroke depression, anxiety, cognitive impairment, MMSE, HADS, PHQ-9, ischemic stroke, hemorrhagic stroke, mental health

## Abstract

**Background:** Stroke represents a leading cause of disability worldwide and is frequently associated with cognitive impairment, anxiety, and post-stroke depression (PSD), all of which can hinder rehabilitation and reduce quality of life. This study aimed to evaluate the correlations between cognitive function, depression, and anxiety in stroke survivors. **Methods:** A total of 71 patients (41 female, 30 male; mean age 68.1 years, range 42–88) were assessed during rehabilitation using the Mini-Mental State Examination (MMSE), the Hospital Anxiety and Depression Scale (HADS), and the Patient Health Questionnaire-9 (PHQ-9). Stroke type and comorbidities, including hypertension, chronic ischemic heart disease, atrial fibrillation, and type II diabetes, were also recorded. **Results:** As expected, most patients experienced ischemic strokes (73.1%), while 16.9% had hemorrhagic strokes. Comorbidities were highly prevalent, particularly hypertension (63 patients) and chronic ischemic heart disease (60 patients). Cognitive impairment (MMSE < 24) was observed in 28.2% of participants. Emotional assessment showed a mean HADS score of 11.55, with 36.6% of patients classified as having moderate to severe depression (PHQ-9 ≥ 10). Hemorrhagic stroke patients reported slightly higher PHQ-9 scores (8.4 compared to 8.2), while ischemic patients had higher HADS scores (11.8 compared to 9.8). A strong correlation was found between PHQ-9 and HADS (r = 0.90), while MMSE scores showed weak associations with emotional outcomes. **Conclusions**: Cognitive and affective disturbances are common during stroke rehabilitation, with depression and anxiety strongly interrelated but only weakly linked to cognitive decline. These findings emphasize the need for integrated screening and mental health support in rehabilitation programs. Future studies may explore technology-assisted tools, including virtual reality, to enhance patient engagement and recovery.

## 1. Introduction

Stroke is a major cause of disability worldwide, with profound implications for both physical and cognitive health [1]. One of the most significant complications following a stroke is the development of post-stroke depression (PSD), a condition that can severely hinder the rehabilitation process [2]. In addition to depression, many stroke survivors experience cognitive decline, which can lead to or exacerbate conditions such as dementia [3]. Understanding the complex relationship between stroke, dementia, and depression is critical, as these conditions not only reduce the quality of life for patients but also significantly impair their chances of achieving successful recovery [4].

Strokes can be classified into two main categories based on the underlying cause. Ischemic strokes, which are the most common, occur when a blood clot blocks a vessel in the brain, cutting off the blood supply to the affected area. This deprivation leads to tissue death and often results in long-term physical and cognitive deficits [5,6]. Hemorrhagic strokes, though less common, are more severe. They are caused by the rupture of a blood vessel in the brain, leading to bleeding and subsequent damage to surrounding tissues. This type of stroke carries a higher risk of mortality and is frequently associated with more significant neurological impairments [7].

Similarly, dementia presents itself in various forms, many of which can be triggered or worsened by stroke [8]. Vascular dementia is often associated not only with stroke and ischemic events, but also with other forms of cerebrovascular pathology, including chronic hypoperfusion, disruption of the blood–brain barrier, and neuroinflammatory processes, which may arise from both sterile and infectious causes [9,10]. It is characterized by challenges in reasoning, problem-solving, and memory [11]. Alzheimer’s disease, though primarily caused by degenerative processes, can also be exacerbated by strokes, which hasten the cognitive decline in individuals already suffering from this condition. In some cases, mixed dementia may occur, where patients experience both vascular dementia and Alzheimer’s disease, making diagnosis and treatment more complex [12].

Post-stroke depression is a common yet underdiagnosed condition that affects approximately one-third of stroke survivors [13]. Depression in this context significantly undermines the rehabilitation process, as patients often become disengaged from therapy and exhibit reduced motivation for physical activity. This lack of compliance with treatment regimens not only delays recovery but also increases the risk of recurrent strokes and worsens cognitive decline [14]. The emotional impact of depression can further lead to the deterioration of social relationships, isolating patients and negatively affecting their overall well-being [15]. Addressing depression in stroke patients is, therefore, a crucial component of their holistic care, as it has a profound impact on the outcomes of their recovery journey [16].

The treatment of post-stroke conditions, including depression and cognitive decline, requires a comprehensive approach involving pharmacological, psychological, and rehabilitative interventions [13,17,18]. Pharmacological treatments, such as antidepressants like selective serotonin reuptake inhibitors (SSRIs), are often prescribed to manage post-stroke depression [18]. Additionally, there is ongoing research into neuroprotective drugs that aim to reduce cognitive decline and promote neural recovery following a stroke [19]. Rehabilitation therapies, including physical therapy, occupational therapy, and cognitive rehabilitation, are essential for helping stroke survivors regain motor functions, improve cognitive abilities, and enhance their overall quality of life [20].

In addition to these traditional approaches, psychological interventions such as cognitive-behavioral therapy (CBT) and counseling have shown significant promise in helping patients cope with the emotional aftermath of a stroke [21]. These therapies provide patients with the tools to manage their depression, increase their motivation for rehabilitation, and develop a more positive outlook on their recovery [22]. When it comes to stroke survivors, correcting previous lifestyle factors such as excessive alcohol consumption, unhealthy dietary patterns, and smoking is essential, as these modifications significantly reduce the risk of recurrent cerebrovascular events and contribute to improved long-term neurological and cardiovascular outcomes [23]. Furthermore, continuous developments in the field of stroke treatment offer hope for more effective interventions. Emerging treatments, such as brain stimulation techniques like transcranial magnetic stimulation (TMS), have shown potential in alleviating symptoms of both post-stroke depression and cognitive decline [24]. Innovative therapies like stem cell therapy and gene therapy are also being actively researched, offering the possibility of personalized and more effective treatments in the near future [25].

In this study, we emphasize the need for routine screening of depression and anxiety during post-stroke rehabilitation, alongside cognitive assessment. Using MMSE, HADS, and PHQ-9, we conducted a cross-sectional analysis to examine the relationship between cognitive performance and affective symptoms in stroke survivors. We hypothesized that depression and anxiety are common and strongly interrelated, but only weakly associated with global cognition and basic clinical variables such as stroke subtype or sex. By clarifying these relationships in a real-world rehabilitation context, this study provides evidence to support mental health–integrated rehabilitation and establishes a rationale for future use of virtual reality (VR) tools to enhance cognitive–emotional assessment and engagement in therapy.

The remainder of this manuscript is organized as follows: Section 2 details the study design, setting, participants, instruments, and statistical analysis; Section 3 presents the results of cognitive and affective assessments and their associations with demographic and clinical variables; Section 4 discusses these findings in the context of prior literature and outlines future directions, including the rationale for VR-based approaches; Section 5 summarizes the main conclusions.

## 2. Materials and Methods

This observational, cross-sectional study was conducted at the Craiova Clinical Hospital of Neuropsychiatry and focused on patients in the post-stroke recovery phase. All assessments were performed between 3 months and 1 year after the stroke event, corresponding to the subacute to early chronic recovery phase. Patients were evaluated when they presented for clinical rehabilitation, ensuring a stable neurological and psychological status suitable for cognitive and emotional screening.

A total of 71 patients with confirmed ischemic or hemorrhagic stroke were included. The inclusion criteria comprised: a confirmed diagnosis of ischemic or hemorrhagic stroke, availability of both clinical and psychometric data, and informed consent for data use in research. Exclusion criteria included the presence of preexisting neurodegenerative conditions, as determined by a combination of clinical and imaging data obtained at hospital admission. Specifically, patient anamnesis, review of prior medical documentation, and neurological assessment were used to identify any known diagnosis of Alzheimer’s disease, Parkinson’s disease, or other forms of dementia. Another exclusion criteria were incomplete clinical or psychometric evaluations. The study was conducted with the approval of the Institutional Ethics Committee (approval no. 2320/26.03.2025).

Demographic and clinical data were systematically collected, including age (years), sex (male/female), stroke type (ischemic, hemorrhagic, or other specified subtypes), and comorbidities such as hypertension, ischemic heart disease, atrial fibrillation, dyslipidemia, and chronic obstructive pulmonary disease (COPD). To capture a multidimensional perspective on post-stroke recovery, cognitive and affective status were assessed using three validated instruments: the Mini-Mental State Examination (MMSE), the Hospital Anxiety and Depression Scale (HADS), and the Patient Health Questionnaire-9 (PHQ-9).

The MMSE is a widely used tool for evaluating global cognitive function. It examines multiple cognitive domains such as orientation, memory, attention, language, and visuospatial skills, resulting in a score between 0 and 30. Higher scores indicate better cognitive performance, while scores below 24 suggest varying degrees of cognitive impairment [26].

The HADS, specifically designed for medical settings, was administered to assess anxiety and depression while minimizing confounding effects from somatic symptoms commonly present after stroke. It consists of 14 items, equally divided into two subscales: HADS-A (anxiety) and HADS-D (depression). Each item is rated on a four-point Likert scale (0–3), producing a score range of 0 to 21 per subscale [27].

The PHQ-9, a self-administered questionnaire based on DSM criteria, was used to evaluate the severity of depression. Its nine items, each rated from 0 to 3, generate a total score ranging from 0 to 27, allowing classification of depression from minimal to severe. Importantly, one item explicitly screens for suicidal ideation, making the PHQ-9 a critical tool for identifying patients at risk [28].

All three instruments were administered during the rehabilitation period. This multimodal approach allowed us to examine the interplay between cognitive impairment, anxiety, and depression in stroke survivors, providing a comprehensive understanding of the challenges these patients face during recovery.

For statistical analysis, descriptive measures (mean, median, standard deviation, frequencies) were first calculated. Continuous variables were summarized using mean ± standard deviation (SD) for normally distributed data and median with interquartile range (IQR) for non-normally distributed data. Categorical variables were summarized as counts (*n*) and percentages (%).

To evaluate group differences, the Mann–Whitney U test was used for sex-based comparisons, while the Kruskal–Wallis test assessed differences between stroke types. Associations between age and psychometric scores were examined using Spearman’s rank correlation because the variables were not normally distributed and represented ordinal or non-linear continuous data, for which non-parametric correlation analysis is more appropriate than Pearson’s r. To determine the simultaneous effect of multiple predictors (age, sex, comorbidities) on MMSE, HADS, and PHQ-9 scores, multivariable linear regression models (ordinary least squares, OLS) were employed. Statistical significance was defined as *p* < 0.05.

In addition to *p*-values, effect sizes were calculated to provide a measure of the magnitude of observed differences independent of sample size. For the Mann–Whitney U tests, the rank-biserial correlation (r) was used, and for the Kruskal–Wallis tests, eta-squared (η^2^) was computed using the formula η^2^ = (*H* − *k* + 1)/(*N* − *k*), where *H* is the Kruskal–Wallis statistic, *k* is the number of groups, and *N* the total sample size. Effect sizes were interpreted using conventional thresholds (small: r < 0.3, η^2^ < 0.06; medium: r ≈ 0.3–0.5, η^2^ ≈ 0.06–0.14; large: r > 0.5, η^2^ > 0.14).

All statistical analyses were conducted using IBM SPSS Statistics, version 29.0 (IBM Corp., Armonk, NY, USA).

During the preparation of this manuscript, the authors used ChatGPT (ScholarAI, GPT-4o model, OpenAI, August 2025) for the purposes of grammar correction, language polishing, graphical concept suggestions, and structuring of scientific content. The authors have thoroughly reviewed and edited all AI-generated outputs and take full responsibility for the final content of this publication. No AI tool was used for generating original research data or conducting formal analyses.

## 3. Results

### 3.1. General Characteristics

The final cohort included 71 patients with a confirmed diagnosis of stroke (Table 1). The mean age was 68.1 years (range: 42–88 years), and the sex distribution comprised 41 women (57.7%) and 30 men (42.3%).

The majority of cases were diagnosed as ischemic or hemorrhagic stroke, while a smaller proportion fell into other subtypes. The “Other” category (9%) includes lacunar infarctions, vertebrobasilar or brainstem ischemic strokes, and subarachnoid hemorrhages. These subtypes were grouped under “Other” due to their low individual frequencies in the cohort, which did not allow for meaningful subgroup analysis. However, they were retained in the overall descriptive statistics to provide a complete representation of stroke etiology within the sample.

Regarding comorbidities, arterial hypertension and ischemic heart disease were the most frequently reported, often coexisting within the same patient, reflecting the typical vascular risk profile of this population.

### 3.2. Distribution of Cognitive and Affective Scores

**Figure 1:**
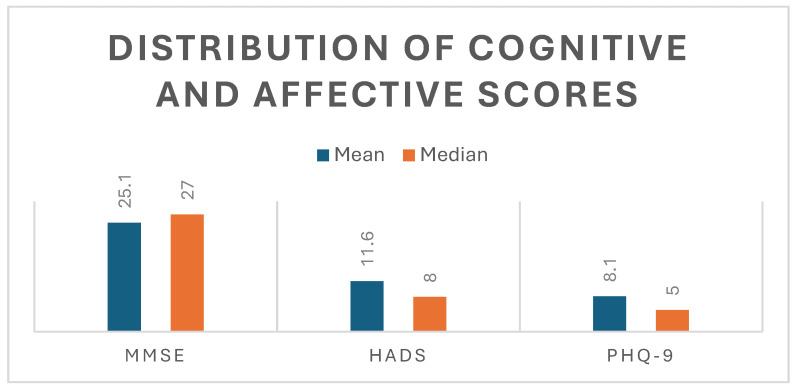
Distribution of cognitive and affective scores. Mean and median values of cognitive (MMSE) and affective (HADS, PHQ-9) scores, illustrating higher central tendency in cognitive performance and greater variability in affective measures.

The standardized psychometric evaluations demonstrated, as presented in Table 2, the following:

These findings indicate a moderate degree of cognitive impairment across the cohort, as well as the presence of clinically relevant affective symptoms, with substantial interindividual variability suggesting heterogeneity in recovery trajectories (Figure 1).

#### Correlation Between PHQ-9 and HADS

A strong and statistically significant positive correlation was found between PHQ-9 and HADS scores (Spearman’s ρ = 0.90, *p* < 0.001), indicating a substantial overlap between depressive and anxiety symptomatology in this cohort. This association supports the concurrent validity of both instruments in assessing affective disturbances in stroke survivors during rehabilitation.

### 3.3. Sex-Based Comparisons

To explore potential sex-related differences in cognitive and affective outcomes, the Mann–Whitney U test was applied, given the non-normal distribution of scores (Table 3). Results indicated no statistically significant differences between men and women:

These results suggest that sex did not exert a measurable influence on either cognitive or emotional outcomes in this cohort.

### 3.4. Stroke-Type Comparisons

**Figure 2:**
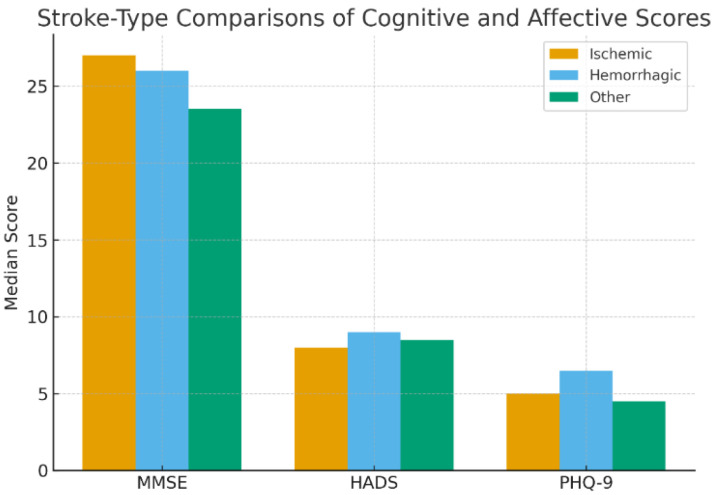
Stroke-type comparisons of cognitive and affective scores. Grouped bar chart illustrating median MMSE, HADS, and PHQ-9 scores across ischemic, hemorrhagic, and other stroke subtypes. The analysis demonstrated no statistically significant differences between groups, indicating that stroke type was not a determinant of cognitive impairment or affective symptom burden in this cohort.

Differences in outcomes between ischemic, hemorrhagic, and other stroke subtypes were evaluated using the Kruskal–Wallis test (Table 4). Across all three psychometric measures, no statistically significant differences were identified:

These results indicate that stroke type was not a determinant of either cognitive impairment or affective burden in the studied sample (Figure 2).

### 3.5. Correlations with Age

**Figure 3:**
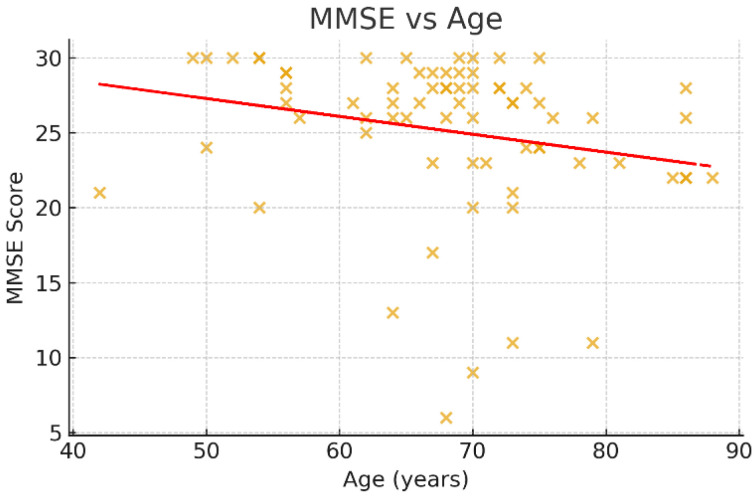
Scatter plot of MMSE scores compared to age. This scatter plot illustrates the relationship between age and cognitive function as measured by the Mini-Mental State Examination (MMSE). Each point represents an individual patient (*n* = 71). A downward trendline indicates a negative association between age and MMSE scores, suggesting that older patients tend to have lower cognitive performance in the post-stroke recovery phase. Although the correlation did not reach statistical significance (Spearman’s *ρ* < 0, *p* ≈ 0.07), the observed trend is consistent with the expected age-related decline in cognitive reserve.

**Figure 4:**
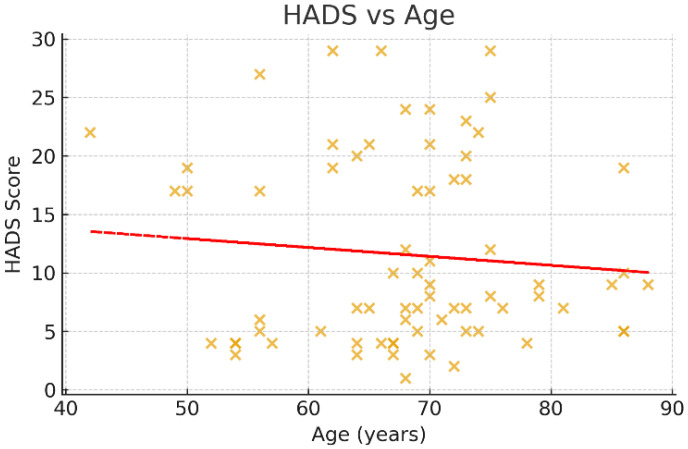
Scatter plot of HADS scores and age. This scatter plot depicts the distribution of Hospital Anxiety and Depression Scale (HADS) total scores across different ages. The red trendline demonstrates a flat trajectory, indicating no meaningful correlation between age and anxiety/depression symptom burden in this sample (Spearman’s ρ ≈ 0, *p* > 0.05). These findings suggest that emotional distress following stroke was independent of patient age, reflecting the heterogeneous nature of affective responses in stroke survivors.

**Figure 5:**
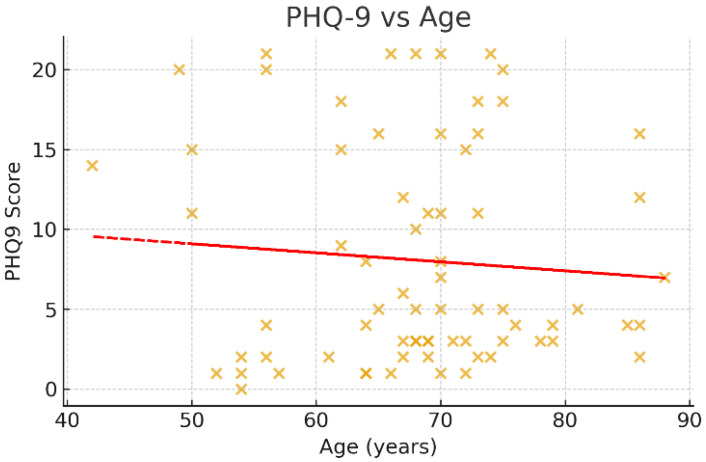
Scatter plot of PHQ-9 scores and age. This figure shows the association between age and depressive symptoms, as measured by the Patient Health Questionnaire-9 (PHQ-9). The scatter points are widely dispersed, and the regression trendline is nearly horizontal, indicating no significant correlation between age and depressive symptom severity (Spearman’s ρ ≈ 0, *p* > 0.05). This suggests that post-stroke depression in this cohort was not age-dependent, further highlighting the role of other psychosocial and clinical factors.

Associations between age and psychometric scores were examined using Spearman’s rank correlation coefficient (Figure 3, Figure 4 and Figure 5). A negative trend was observed between age and MMSE (ρ < 0, *p* ≈ 0.07), suggesting that older patients tended to score lower on cognitive assessments, although this did not reach conventional statistical significance. No significant correlations were observed between age and HADS or PHQ-9 scores (*p* > 0.05).

### 3.6. Multivariable Regression

**Figure 6:**
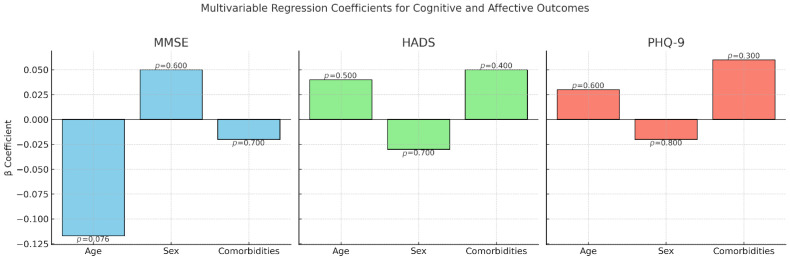
Multivariable regression coefficients for cognitive and affective outcomes. Bar charts display β coefficients for age, sex, and comorbidity status obtained from multivariable linear regression models (ordinary least squares). Three outcomes were assessed: MMSE (left), HADS (center), and PHQ-9 (right). For MMSE, age demonstrated a marginally significant negative association (*β* = −0.117, *p* = 0.076), suggesting lower cognitive performance with increasing age. No significant associations were found for sex or comorbidities in relation to MMSE, HADS, or PHQ-9 (*p* > 0.05). These findings indicate that age may play a role in post-stroke cognitive decline, whereas affective outcomes appear independent of the demographic and clinical predictors analyzed.

To further evaluate the joint influence of age, sex, and comorbidity status, multivariable linear regression models (ordinary least squares, OLS) were constructed (Table 5 and Figure 6).

For the purpose of regression analyses, the variable “Comorbidities” was operationalized as a binary indicator (0 = absence of comorbidities; 1 = presence of one or more comorbid conditions). This dichotomization was applied to reduce model complexity and avoid multicollinearity, given that most patients presented overlapping cardiovascular risk factors.

For MMSE, age demonstrated a marginally significant negative effect (β = −0.117, *p* = 0.076), while sex and comorbidities were not statistically significant predictors.

For HADS and PHQ-9, none of the included predictors reached significance (*p* > 0.05).

These models indicate that age may play a role in cognitive decline post-stroke, whereas affective outcomes appear to be independent of the demographic and clinical variables assessed in this study.

#### Effect Size Analysis

To complement the non-parametric comparisons, we calculated effect sizes to aid the interpretation of non-significant findings. For Mann–Whitney U tests (sex comparisons), the rank-biserial correlation (r) was used, while for Kruskal–Wallis tests (stroke type comparisons), eta-squared (η^2^) was computed.

As shown in Table 6, effect sizes were small across all analyses (|r| < 0.2; η^2^ < 0.3), indicating that the lack of statistical significance is likely due to a true absence of large group effects rather than to limited statistical power.

These findings reinforce the interpretation that demographic and stroke-type factors exert limited influence on cognitive and affective outcomes in this cohort.

## 4. Discussion

The present study investigated cognitive and affective outcomes in a cohort of 71 patients undergoing post-stroke rehabilitation, with particular attention to the influence of demographic and clinical factors such as age, sex, stroke type, and comorbidities. Using standardized psychometric tools (MMSE, HADS, PHQ-9) and a robust statistical approach, our findings provide insight into the determinants of post-stroke recovery in cognitive and emotional domains.

The overall profile of our cohort was consistent with the epidemiology of stroke: mean age of 68.1 years, a predominance of ischemic stroke, and a high prevalence of vascular comorbidities such as hypertension and ischemic heart disease. These characteristics reflect the typical vascular risk burden observed in stroke populations worldwide and underline the external validity of our sample.

With respect to cognitive and emotional status, the mean MMSE score of 25.1 suggests a moderate degree of cognitive impairment, while HADS and PHQ-9 scores indicated the presence of anxiety and depressive symptoms in a substantial subset of patients. Importantly, the variability in these measures was considerable, emphasizing the heterogeneous nature of post-stroke recovery and the need for individualized approaches in rehabilitation.

When stratified by sex, no statistically significant differences were found across MMSE, HADS, or PHQ-9 scores. While some prior studies have reported higher rates of depression or worse cognitive outcomes among women following stroke, our results did not confirm these associations [29,30]. This may be due to sample size, but it also highlights the complexity of psychosocial and biological factors that mediate sex differences in stroke recovery. Similarly, stroke subtype (ischemic, hemorrhagic, and other) was not associated with significant differences in cognitive or affective outcomes, suggesting that lesion type alone is not sufficient to predict recovery trajectories. These findings echo recent literature indicating that post-stroke cognition and mood are determined more by lesion location, network disruption, and psychosocial context than by gross stroke classification [31].

Age, on the other hand, showed a clear trend toward a negative association with cognitive outcomes. Older patients had lower MMSE scores, with a marginally significant effect in multivariable regression analysis (β = −0.117, *p* = 0.076). Although this did not cross the conventional significance threshold, the directionality is consistent with the well-established concept that aging reduces cognitive reserve and neuroplasticity, thereby limiting recovery potential after brain injury. Interestingly, age was not related to affective outcomes, as both HADS and PHQ-9 scores showed flat associations with age. This suggests that emotional recovery and vulnerability to depression or anxiety may depend more on psychosocial support, coping strategies, and rehabilitation environments rather than chronological age.

The multivariable regression models further reinforced these findings: age was the only variable to show a marginal effect on cognition, while sex and comorbidities did not significantly influence outcomes. These results highlight the limitations of using broad demographic or clinical predictors alone to stratify post-stroke patients. Instead, more granular assessments, such as lesion topography, biomarkers of inflammation and neuroplasticity, and psychosocial context, may be necessary to better predict outcomes [32,33].

Taken together, our findings contribute to the growing evidence that post-stroke rehabilitation should not rely solely on traditional demographic or stroke-type stratification. Instead, comprehensive screening for both cognitive and affective impairments using validated tools should be standard practice. Even in the absence of clear predictors, routine assessment ensures early identification of patients at risk and allows tailored interventions.

Several limitations of our study must be acknowledged. The relatively small sample size and single-center design limit the generalizability of findings. Furthermore, the cross-sectional nature of our assessment precludes conclusions about the trajectory of recovery over time. Longitudinal, multicenter studies incorporating multimodal data, clinical, imaging, and biological, are warranted to refine predictive models. Nonetheless, the strengths of our study include the use of validated psychometric instruments, rigorous statistical analysis, and the integration of cognitive and affective outcomes in a unified framework.

In conclusion, our results underscore the heterogeneity of post-stroke recovery, with age emerging as a potential risk factor for cognitive decline but not for affective symptoms. Sex, stroke type, and comorbidities were not predictive of outcomes. These findings reinforce the importance of systematic, multidimensional assessment of all stroke survivors, regardless of demographic or clinical profile, to optimize rehabilitation strategies.

An additional aspect that deserves consideration is the interaction between cognitive and motor outcomes in post-stroke rehabilitation. Although our study did not directly assess motor recovery, the literature increasingly supports the view that cognitive status is a strong determinant of motor rehabilitation potential [34]. Motor learning after stroke relies on cognitive resources such as attention, working memory, and executive function, which facilitate the understanding of movement strategies, error correction, and adaptation to new motor tasks [35]. Patients with preserved cognitive abilities have been shown to achieve better functional recovery of both upper and lower limbs, perform more intensive motor training, and maintain higher levels of independence in activities of daily living. Conversely, individuals with cognitive impairment often exhibit slower motor progress, reduced rehabilitation engagement, and poorer functional outcomes despite similar motor deficits at baseline. This suggests that cognitive impairment may act as a hidden barrier in rehabilitation, limiting the capacity to benefit from repetitive task practice and physiotherapy [36].

Post-stroke rehabilitation should adopt an integrated neurorehabilitation model in which cognitive screening is performed systematically, and targeted cognitive interventions are incorporated alongside motor therapy. Approaches such as dual-task training, task-specific learning combined with executive function exercises, or technology-based rehabilitation (such as virtual reality and neurofeedback) have shown promising results in enhancing both cognitive and motor outcomes.

### 4.1. Future Direction

An important direction for the future lies in the integration of innovative technologies into post-stroke care. Among these, virtual reality (VR) has gained increasing attention as a tool capable of combining physical, cognitive, and affective rehabilitation in an engaging and patient-centered manner [37]. VR-based interventions can provide immersive and repetitive training environments, deliver real-time feedback, and simulate real-life tasks that challenge not only motor but also cognitive and emotional domains [38]. Recent studies have shown encouraging results, with VR improving motor function, balance, and aspects of cognition such as attention and memory, while also reducing anxiety and depressive symptoms by enhancing motivation and participation [39].

In addition to its rehabilitative potential, we envision VR evolving into a platform powered by artificial intelligence (AI) capable of conducting comprehensive, real-time assessments of cognitive, emotional, and functional status [40]. By integrating AI algorithms into VR systems, it becomes feasible to automate and dynamically evaluate clinical scales, such as MMSE, HADS, and PHQ-9, beginning at hospital admission and continuing throughout the recovery process [41]. Using a single VR headset, patients could undergo immersive, standardized evaluations that monitor neurocognitive and affective trajectories without requiring extensive clinical manpower [42]. Similar AI-driven rehabilitation platforms have already shown promise in Parkinson’s and Alzheimer’s disease, offering adaptive, personalized feedback to support neural recovery [41]. Extending such technology to stroke care could enable earlier detection of psychological vulnerability, optimize therapeutic strategies, and ultimately foster a more responsive and efficient rehabilitation ecosystem.

Given the absence of strong demographic or clinical predictors for affective outcomes in our cohort, we believe that VR could serve not only as a rehabilitative tool but also as a novel platform for cognitive–affective assessment [43]. Its capacity to capture dynamic performance metrics, patient engagement, and emotional responses during training opens new avenues for personalized monitoring [44]. Unlike static clinical assessments, VR-based environments generate continuous data streams that reflect how patients adapt to cognitive load, time pressure, and environmental complexity in real time. These metrics can also be complemented by emotion-sensitive indicators such as task avoidance, error recovery time, and response consistency, and can be integrated with physiological monitoring (eye tracking, heart rate variability) to infer emotional states [44]. Future work by our team will explore the use of VR-based paradigms for early identification of cognitive and emotional vulnerability post-stroke, aiming to complement standard psychometric tools and contribute to more tailored rehabilitation strategies.

### 4.2. Sociodemographic Considerations and the Value of Scalable Technologies

A unique feature of our cohort is that many patients originate from socioeconomically disadvantaged regions with a higher incidence of stroke and limited access to specialized care. In such settings, quality of life is often diminished, and early, efficient rehabilitation services are difficult to implement. The integration of low-cost, scalable technologies, such as AI-powered VR platforms, could provide immense benefits by offering standardized assessments and therapy even in resource-limited environments. Their ease of deployment and minimal training requirements make them particularly suitable for healthcare systems in countries facing similar structural and financial constraints.

### 4.3. The Role of Psychiatric History in Stroke Recovery

Another important but often overlooked factor is the presence of preexisting psychiatric conditions, particularly depression or anxiety. Patients with a history of mental illness may be more vulnerable to post-stroke affective disturbances, which can negatively impact rehabilitation outcomes. Future studies should investigate this connection, as identifying at-risk individuals early could enable targeted interventions to improve recovery trajectories.

## 5. Conclusions

This study provides an integrated analysis of cognitive and affective outcomes in stroke survivors undergoing rehabilitation, using standardized instruments such as the MMSE, HADS, and PHQ-9. Our findings highlight that while moderate cognitive impairment and affective symptoms are common in this population, demographic and clinical factors such as sex, stroke type, and comorbidities were not predictive of outcome variability. Age emerged as the only factor showing a marginal association with cognitive performance, underscoring its role as a determinant of reduced neuroplasticity and cognitive reserve in post-stroke recovery.

In this cohort, we did not find evidence that sex, stroke type, or comorbidities significantly influenced cognitive or affective outcomes. However, given the modest sample size, these null findings should be interpreted cautiously, as smaller effects may not have been detectable within the current study design.

These results emphasize the need for systematic screening of all stroke survivors, regardless of demographic or clinical profile, to ensure early detection and management of cognitive and affective sequelae. Reliance on traditional clinical predictors alone appears insufficient, and rehabilitation strategies must adopt a multidimensional, individualized approach.

Looking ahead, innovative technologies such as VR and AI hold promise for both assessment and rehabilitation. By combining motor, cognitive, and emotional training in immersive environments, VR could complement conventional tools and provide more dynamic insights into patient functioning. Our team aims to further explore the integration of VR-based paradigms into post-stroke care as a future research direction.

## Figures and Tables

**Table 1:** Baseline characteristics of the study cohort (*n* = 71).

Variable	Value
Age (years)	68.1 ± 10.4 (range 42–88)
Sex, *n* (%)	Female: 41 (57.7%), Male: 30 (42.3%)
* Stroke type, *n* (%)	Ischemic: 40 (56.3%), Hemorrhagic: 25 (35.2%), Other: 6 (8.5%)
Comorbidities, *n* (%)	Hypertension (HTA): 63 (88.7%) Ischemic heart disease (CIC): 60 (84.5%) Other comorbidities †: 28 (39.4%)
MMSE	27 (IQR 23–29)
HADS total score	8 (IQR 5–17)
PHQ-9 score	5 (IQR 3–16)

* Other stroke subtypes include lacunar infarctions, vertebrobasilar ischemia, and subarachnoid hemorrhage. † Includes atrial fibrillation, diabetes mellitus type II, dyslipidemia, COPD, obesity, chronic liver disease, chronic kidney disease, and malignancy. Values are presented as mean ± standard deviation (SD) for normally distributed variables and median with interquartile range (IQR) for non-normally distributed variables.

**Table 2:** Distribution of cognitive and affective scores in the study cohort.

	Mean	Median
MMSE	25.1	27
HADS	11.6	8
PHQ-9	8.1	5

Mean and median values are presented for MMSE, HADS, and PHQ-9. Results indicate moderate cognitive impairment (MMSE) and the presence of clinically relevant affective symptoms (HADS, PHQ-9), with variability across patients.

**Table 3:** Sex-based comparisons of MMSE, HADS, and PHQ-9 scores.

Scale	Median (Men)	Median (Women)	*p*-Value
MMSE	27	26	0.297
HADS	7	9	0.278
PHQ-9	4.5	6	0.448

Median values are presented for men and women, with *p*-values obtained from the Mann–Whitney U test. No statistically significant differences were observed between sexes across cognitive (MMSE) or affective (HADS, PHQ-9) outcomes (*p* > 0.05).

**Table 4:** Stroke-type comparisons of MMSE, HADS, and PHQ-9 scores.

Scale	Ischemic (Median)	Hemorrhagic (Median)	Other (Median)	*p*-Value
MMSE	27	26	23.5	0.147
HADS	8	9	8.5	0.839
PHQ-9	5	6.5	4.5	0.967

Median values are reported for ischemic, hemorrhagic, and other stroke subtypes, with *p*-values obtained from the Kruskal–Wallis test. No statistically significant differences were identified across cognitive (MMSE) or affective (HADS, PHQ-9) outcomes (*p* > 0.05).

**Table 5:** Multivariable regression analysis of predictors for cognitive and affective outcomes.

Predictor	MMSE (β)	MMSE (*p*-Value)	HADS (β)	HADS (*p*-Value)	PHQ-9 (β)	PHQ-9 (*p*-Value)
Age	−0.117	0.076	0.04	0.5	0.03	0.6
Sex	0.05	0.6	−0.03	0.7	−0.02	0.8
Comorbidities	−0.02	0.7	0.05	0.4	0.06	0.3

β coefficients and corresponding *p*-values are reported for age, sex, and comorbidity status across MMSE, HADS, and PHQ-9 outcomes. Age demonstrated a marginally significant negative association with MMSE (*β* = −0.117, *p* = 0.076), suggesting lower cognitive performance with increasing age. No significant associations were observed for sex or comorbidities in relation to any of the assessed outcomes (*p* > 0.05).

**Table 6:** Effect size analysis for non-parametric comparisons.

Comparison	Variable	Test	*p*-Value	Effect Size	Interpretation
Sex	MMSE	Mann–Whitney U	0.183	r = 0.19	Small effect
	HADS	Mann–Whitney U	0.161	r = −0.20	Small effect
	PHQ-9	Mann–Whitney U	0.418	r = −0.12	Very small effect
Stroke Type	MMSE	Kruskal–Wallis	0.308	η^2^ = 0.26	Small–medium effect
	HADS	Kruskal–Wallis	0.536	η^2^ = −0.09	Negligible
	PHQ-9	Kruskal–Wallis	0.644	η^2^ = −0.25	Small–medium, non-significant

## Data Availability

Dataset available on request from the authors.

## Abbreviations

The following abbreviations are used in this manuscript:

AI	Artificial Intelligence
CBT	Cognitive-Behavioral Therapy
COPD	Chronic Obstructive Pulmonary Disease
DSM	Diagnostic and Statistical Manual of Mental Disorders
HADS	Hospital Anxiety and Depression Scale
HADS-A	Hospital Anxiety and Depression Scale-Anxiety Subscale
HADS-D	Hospital Anxiety and Depression Scale-Depression Subscale
MMSE	Mini-Mental State Examination
OLS	Ordinary Least Squares
PHQ-9	Patient Health Questionnaire-9 Items
PSD	Post-Stroke Depression
SPSS	Statistical Package for the Social Sciences
SSRIs	Selective Serotonin Reuptake Inhibitors
TMS	Transcranial Magnetic Stimulation
VR	Virtual Reality
